# Vitamin D Deficiency Exacerbates Poor Sleep Outcomes with Endocrine-Disrupting Chemicals Exposure: A Large American Population Study

**DOI:** 10.3390/nu16091291

**Published:** 2024-04-26

**Authors:** Ruiqi Zhou, Zhongwen Chen, Tingting Yang, Huiwen Gu, Xiaohong Yang, Shuqun Cheng

**Affiliations:** Department of Occupational and Environmental Health, School of Public Health, Chongqing Medical University, Chongqing 400016, China; 2022110612@stu.cqmu.edu.cn (R.Z.); 2021120755@stu.cqmu.edu.cn (Z.C.); tingtingyang@stu.cqmu.edu.cn (T.Y.); guhuiwen@stu.cqmu.edu.cn (H.G.); 2021120810@stu.cqmu.edu.cn (X.Y.)

**Keywords:** vitamin D, sleep, environmental endocrine disruptors, NHANES

## Abstract

Phthalates and bisphenol A are recognized as the predominant endocrine-disrupting substances (EDCs) in the environment, but their impact on sleep health remains unclear. Vitamin D has often been reported to play a role in sleep health and may be affected by endocrine-disrupting compounds. The study utilized data from 5476 individuals in the NHANES project to investigate the correlation between combined exposure to environmental EDCs and sleep duration through modeling various exposures. Furthermore, it emphasizes the importance of vitamin D in the present scenario. Preliminary analyses suggested that vitamin D-deficient individuals generally slept shorter than individuals with normal vitamin D (*p* < 0.05). Exposure to Mono-ethyl phthalate (MEP), triclosan (TRS), and Mono-benzyl phthalate (MZP), either alone or in combination, was associated with reduced sleep duration and a greater risk of vitamin D deficiency. Individuals with low vitamin D levels exposed to TRS experienced shorter sleep duration than those with normal vitamin D levels (*p* < 0.05). TRS and MZP were identified as crucial factors in patient outcomes when evaluating mixed exposures (*p* < 0.05). The results provide new data supporting a link between exposure to EDCs and insufficient sleep length. Additionally, they imply that a vitamin D shortage may worsen the sleep problems induced by EDCs.

## 1. Introduction

Over 1000 EDCs have been identified in the environment, including a range of phenols, industrial chemicals, pesticides, and plasticizers. These compounds are commonly used in modern life and represent a major threat to human health owing to the ease of potential exposure thereto [1]. EDCs can disrupt the production, regulation, and metabolism of hormones, contributing to altered circadian rhythms and a range of adverse sleep-related outcomes [2,3]. BPA and phthalates are the most common EDCs, functioning as endocrine disruptors that modulate sex hormones and impact the hypothalamic-pituitary-adrenergic axis to contribute to the incidence of a range of sleep issues [4,5]. BPA is also among the most widely produced chemicals, with an estimated output in 2022 alone of 6000 kilotons [6], leading to the inevitable exposure of the general public to BPA in the air, water, and food they consume. While alternative chemicals are available, global phthalate use still exceeds 11 tons per minute, with a forecasted use of 84.8 million tons in 2024 [7]. Many of these EDCs are present at high levels in everyday household products as components of food packaging, antifungal agents, or preservatives, resulting in the potential for exposure via ingestion, skin contact, or inhalation [8]. Both the US Environmental Protection Agency and the European Union have, thus, categorized these compounds as priority control pollutants [4,9], and there has been growing research interest in understanding the severe effects of BPA and phthalates on sleep-related health.

Sleep disorders are conditions that interfere with the duration, quality, or timing of sleep, sleep-related behaviors, and physiological characteristics [10], with sleep disorders affecting an estimated 50% of the global population [11]. Despite being highly prevalent, sleep disorders and sleep deprivation are often not regarded as important health issues by the general public. Poor sleep quality, however, can contribute to the incidence of a range of chronic conditions, including endocrine issues, obesity, alcohol abuse, anxiety, diabetes, hypertension, cardiovascular diseases, and neurological or immunological disturbances [12]. Many reports focused on animal-model systems have found that Bisphenol A (BPA) and phthalate exposure can damage hypothalamic neurons in the brain, interfering with the ability of this region to regulate circadian rhythms. These compounds can also compete with endocrine hormones for binding to sex-hormone receptors, resulting in the manifestation of sleep disorders of varying severity [13,14,15,16]. Only a limited number of epidemiological studies conducted to date have examined associations between chemical exposures and poor sleep outcomes. Studies performed in Mexico, for example, documented a relationship between BPA and phthalate exposure and longer sleep duration, earlier sleep timing, and greater sleep fragmentation [17,18]. However, these studies only focused on relatively limited patient populations.

Vitamin D is a steroid hormone consumed through dietary or supplemental sources, in addition to being generated upon the exposure of the skin to UVB radiation. Vitamin D has primarily been studied in the context of musculoskeletal health [19], but there is growing evidence suggesting a link between vitamin D metabolism and a range of sleep-related health outcomes [20,21,22]. One prospective analysis suggested an association between 1,25(OH)_2_D_3_ and overall sleep patterns and the overall incidence of type 2 diabetes [23]. Another interventional analysis focused on individuals > 60 years of age detected a positive correlation between serum levels of 1,25(OH)_2_D_3_ and sleep duration. No reports to date, however, have examined the impact of vitamin D on the effects of BPA and phthalates on sleep disorders.

While previous studies have primarily focused on the correlation between single EDCs and sleep, the present study was conducted by selecting six different statistical approaches (Linear and logistic regression models, the Elastic net [ENET] model, the weighted quantile sum [WQS] regression model, the Quantile G-computation [QGC] model, and the Bayesian Kernel Machine Regression [BKMR] model) to evaluate the association between exposures to chemicals and sleep in adults participating in the U.S. National Health and Nutrition Examination Study (NHANES). In addition, there are fewer epidemiological studies between EDC and vitamin D, and vitamin D may mitigate the health damage caused by EDC. So, this study also analyzes the potential correlation between vitamin D deficiency and EDC-induced sleep disorders to provide some insights into the psychoneurological aspects of EDC-induced damage.

## 2. Materials and Methods

### 2.1. Study Design and Participant

**Figure 1:**
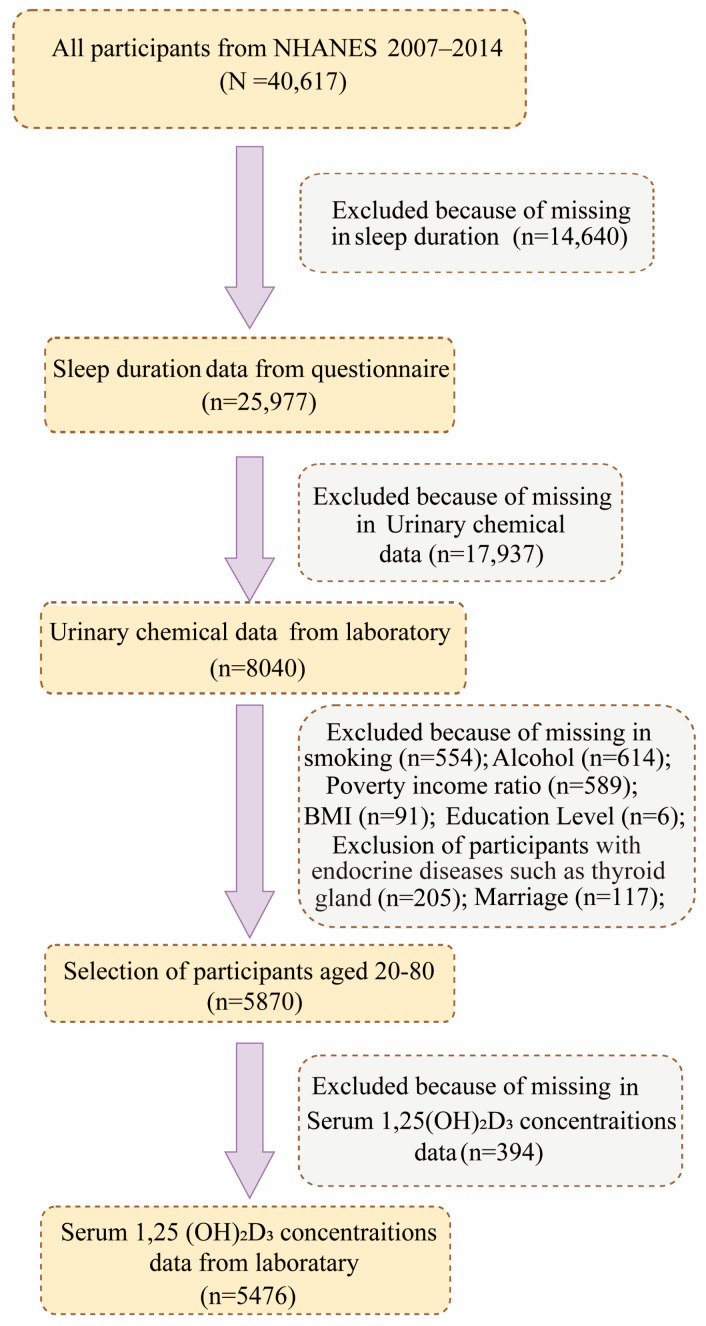
Flowchart of the participants included in the final analysis.

The NHANES research is an official survey undertaken by qualified experts to evaluate the health and nutrition of the overall U.S. population. Informed consent was obtained from all individual participants included in the study. The NHANES agreement has been reviewed and approved by the NCHS Research Ethics Committee. All participants provided written informed consent before participating. Publicly accessible data from four NHANES cycles (2007–2014) were utilized for the current analysis. Participants who did not have data on sleep duration or failed to respond were eliminated from the study (*n* = 14,640). The NHANES survey used a voluntary collection of participant samples, eliminating 17,937 research individuals who did not undergo urine BPA and phthalate testing. To ensure the accuracy of the results, 2170 individuals who lacked data on relevant covariates affecting the results were excluded, including alcohol intake, smoking behavior, thyroid-related diseases, physical activity level, race, marital status, education level, household income/poverty, body mass index (BMI), endocrine-disease status, and age (<20 years or >80 years). Of the remaining participants, 394 with vitamin D-related data deficiencies were excluded, and the remaining 5476 participants were retained for these analyses. The specific screening process is shown in Figure 1.

### 2.2. Exposure Information

BPA and phthalates were selected as common environmental endocrine disruptors for evaluation in this study [24,25]. The urine samples underwent further examination for environmental endocrine disruptors at the National Center for Environmental Health using high-performance liquid chromatography (HPLC) combined with electrospray ionization tandem mass spectrometry (ESI-MS). When levels of these chemicals were below the lowest limit of detection (LOD), the values were replaced with the LOD divided by the square root of 2 as per NHANES laboratory requirements. Professionals supervised the entire detecting process to ensure quality control.

### 2.3. Sleep Time Survey

Sleep-related data in the NHANES survey were derived from the Munich Chronotype Questionnaire (MCTQ) [26], with the self-reported responses to the following question regarding daily sleep: “How much sleep did you get (in hours)?”. Interviewers ask questions at home using the CAPI method. The system’s sample of responders ranges in age from 16 to 150. The study’s participants ranged in age from 20 to 80. To ensure quality control, the questionnaire survey process is carried out by qualified personnel.

### 2.4. Serum 1,25(OH)_2_D_3_ Analyses

Cryopreserved blood samples from study participants were obtained following the NHANES Laboratory Procedures Manual, and serum 1,25(OH)_2_D_3_ levels therein were measured via ultra-high-performance liquid chromatography tandem mass spectrometry (UHPLC-MS/MS). Vitamin D deficiency was defined as 1,25(OH)_2_D_3_ <30 nmol/L (12 ng/mL) [27,28]. Professionals handled the entire detecting process for quality control.

### 2.5. Assessment of Covariates

Participants were classified into three age groups, including young (20–39 years), middle aged (40–59 years), and older (60–80 years) participants [29]. Other evaluated covariates included gender (male and female), ethnicity (non-Hispanic white, non-Hispanic black, Mexican-American, other Hispanic, and other), education level (<9th grade, 9th–11th grade, high school graduate, partial college or related degree, and college graduate or higher), family-income poverty ratios (<1.30, 1.30–3.50, and >3.50 [30]), BMI as measured by trained technicians (underweight [<18.5 kg/m^2^], normal [18.5–24.9 kg/m^2^], overweight [25–29.9 kg/m^2^], and obese [≥30.0 kg/m^2^]) [31], smoking status (nonsmoker [<100 lifetime cigarettes] and smoker), drinking status (<12 alcoholic beverages/year and 12^+^ alcoholic beverages/year). When assessing drinking status, one alcoholic beverage is defined as one drink as 12 oz, one beer as 5 oz, and one glass of wine as 1.5 oz [32] and physical activity (regular activities and infrequent activities).

### 2.6. Statistical Analysis

The continuous variables were expressed as mean ± standard deviation (Mean ± SD) or median (interquartile range (M, IQR)), and the categorical variables were expressed as number of cases (n) and percentage (%). We applied the Rao–Scott chi-square test and Student’s *t*-test based on sampling weights to compare the categorical and continuous characteristics between participants.

Correlations among different urinary EDCs were evaluated with Speraman’s correlation coefficients. In this study, the elastic net model, the generalized linear model, the WQS regression model, the Quantile g-computation model, and Bayesian kernel machine regression were used to analyze the correlation between environmental endocrine disruptors and sleep duration. Regression analyses are reported as coefficients with corresponding 95% CI, and a two-tailed *p* < 0.05 served as the threshold of significance.

All statistical analyses were conducted with R 4.3.2 (R Development Core Team), and the WQS, QGC, and BKMR were implemented with the respective “gWQS”, “Qgcomp”, and “ BKMR” R packages.

#### 2.6.1. Elastic Net Model and Generalized Linear Model

ENET introduces penalty coefficients 1 and 2 based on the linear regression results, enabling the selection of variables by leveraging the advantages of LASSO and ridge regression strategies [33]. In this study, ENET was used to quickly screen EDC metabolites related to sleep time from 15 target compounds, and these relationships were quantified according to the corresponding beta coefficient (β) values. Linear and logistic regression analysis can quickly and easily get the results. The effects of individual or multiple EDCs on sleep duration were assessed with linear and logistic regression analyses. A crude model (Model 1) was not adjusted for any covariates for these analyses. In contrast, Model 2 was adjusted for age, gender, ethnicity, education level, BMI, drinking status, smoking status, and physical activity levels. Both models were adjusted for urinary creatinine levels, as noted above.

#### 2.6.2. WQS Regression Model and Quantile g-Computation Model

WQS regression analyses were used to evaluate the effects of mixtures of compounds on sleep duration, as this model enables the examination of the impact of simultaneous exposure to multiple pollutants on particular health outcomes and can effectively deal with the high collinearity of these substances [34]. In this model, the overall effects of exposure to EDCs on sleep were assessed by establishing a weighted WQS index included in the regression model. This WQS model assumes that each environmental exposure is directional, homogeneous, and linear to the outcome of interest [35]. In view of this inherent limitation, we also use the g calculation method based on quartiles. This model can reflect the correlation between chemical substances in different directions and health outcomes in the same figure. Based on the adaptability of the g calculation, its calculation speed is faster than WQS and BKMR [36].

#### 2.6.3. Bayesian Kernel Machine Regression

The BKMR approach, widely employed in epidemiologic studies, focuses on the effects of mixed environmental exposures, given that it can readily and flexibly assess the combined effects of multiple chemicals with potential non-linear or nonadditive effects [37]. For this study, the following kernel machine regression was employed:

$$Y_{i}=h\left(Z_{i1},\ldots ,Z_{iM}\right)+x_{i}\beta +\varepsilon_{i}$$

where $Y_{i}$ represents the health outcome, $Z_{i}$ corresponds to the chemical exposures, $x_{i}$ denotes potential confounders, $\varepsilon_{i}$ is the residual complying with the normal distribution N (0, δ2), i refers to the individual (i = 1, 2, 3 … n), and h ( ) is an exposure–response function based on non-linear interactions among mixture components. For this study, the MCMC method was used to run this model for 5000 iterations, and the BKMR model was used to calculate posterior inclusion PIPs for each substance, with values exhibiting values closer to 0 considered less important. Mixed exposure effect plots evaluated the mixture’s relevance to the health result. Univariate exposure–response curves have been developed to investigate potential non-linear relationships between substances and outcome indicators while keeping all other substances at the 50th percentile. Bivariate exposure–response curves were also drawn.

## 3. Results

### 3.1. Population Characteristics

In total, 40,617 individuals participated in the NHANES survey from 2007 to 2014. Ultimately, 5476 eligible participants were included in this study based on the inclusion–exclusion criteria shown in Table 1. These included 2709 (48.8%) males and 2767 (51.2%) females, with a mean age of 47.2 ± 16.8 years. Additionally, the majority of participants were non-Hispanic whites (70.4%), well-educated (83.7% above high school), smokers (44.3%), alcohol drinkers (78.7%), and physically active (54.6%). The mean BMI and sleep duration of the study population were 28.9 ± 6.8 kg/m^2^ and 6.8 ± 1.3 h, respectively. About 4% (470) of participants had a vitamin D deficiency.

### 3.2. Distribution, Correlation, and Selection of Environmental Endocrine Disruptors in Urine

**Figure 2:**
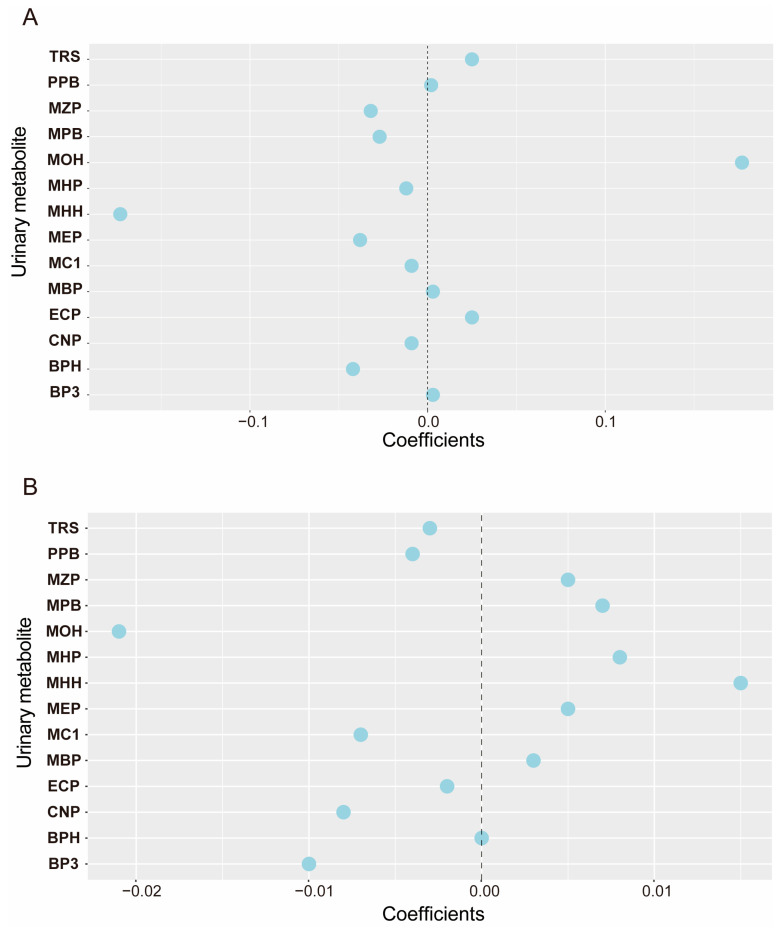
**The correlation coefficients between environmental endocrine disruptors and sleep duration and vitamin D concentration.** The dotted line represents 0, and each circle represents each EDCs substance. The farther the circle is from the dotted line, the greater the correlation between EDCs and sleep duration and vitamin D concentration. The model was adjusted for urinary creatinine, age, gender, race, education level, marital status, family-income-to-poverty ratio, BMI, smoking status, alcohol status, and physical activity. (**A**) Correlation between mixed EDCs metabolites and sleep duration; (**B**) Correlation between mixed EDCs metabolites and vitamin D concentration.

The detectable rates of most urinary phthalate metabolites were above 90%, except for Mono-(2-ethyl)-hexyl phthalate (MHP) and TRS, which had detectable rates of 68.12% and 75.66%, respectively. The median concentration of EDC metabolites in urine was 4.5 (24.84, 197.19) μg/mmol Cr for MEP. The mean values and distributions of other EDC metabolites are summarized in Supplementary Table S1. Furthermore, we performed Spearman’s correlation analysis to find associations among 15 EDC metabolites in urine. We found that the range of Spearman correlation coefficients was in the same direction (*p* < 0.05) for all substances except for Mono (carboxyisoctyl) phthalate (COP) (Supplementary Figure S2), indicating correlations among all substances except COP. The strongest correlation was observed between Mono-(2-ethyl-5-hydroxyhexyl) phthalate (MHH) and Mono-(2-ethyl-5-oxohexyl) phthalate (MOH) (r = 0.97). Here, the results, shown in Supplementary Table S2, revealed a statistically significant correlation between sleep duration and EDCs in the ‘Vitamin D normal’ and ‘Vitamin D deficient’ groups. Additionally, the distribution of EDCs in the body and their correlations were related to the vitamin D status of the participants (Supplementary Figure S2 and Table S3). Subsequently, we used the ENET method to identify fifteen relevant elements in the mixture of EDC metabolites that play an essential role in sleep time. Among the 15 EDCs that were associated with sleep duration, 12 showed a strong association, except Propyl paraben (PPB), Mono-n-butyl phthalate (MBP), and Benzophenone-3 (BP3) (Figure 2). Similarly, we screened for EDC metabolites associated with the risk of vitamin D deficiency using the ENET method and found that, except for Bisphenol A (BPH), the remaining 14 substances were associated with vitamin D deficiency (*p* < 0.05).

### 3.3. Correlation between Urinary EDC Metabolites and Sleep Duration

The results of univariate and multiple linear regression models designed to assess the correlation between EDC metabolites and sleep duration are listed in Table 2. The results of univariate linear regression analysis revealed that, among EDC metabolites, TRS (β: 0.027, 95% CI: (0.000, 0.040), *p* < 0.05) was positively correlated with sleep duration regardless of covariates adjustment, while MEP (β: −0.050, 95% CI: (−0.073, −0.022), *p* < 0.05), MZP (β: −0.034, 95% CI: (−0.085, −0.085), *p* < 0.05), and BPH (β: −0.040, 95% CI: (−0.104, −0.020), *p* < 0.05) were negatively correlated with sleep duration. In addition, after adjusting for covariates, BP3 was positively correlated with sleep duration (*p*< 0.05), and Methyl paraben (MPB) was negatively correlated with PPB and sleep duration (*p*< 0.05).

The results of the multiple linear regression model revealed that urinary MOH (β: 0.186, 95% CI: (0.108, 0.424), *p* < 0.05) and TRS (β: 0.029, 95% CI: (0.001, 0.041), *p* < 0.05) transformed were significantly positively correlated with sleep time with adjusted covariates, while MEP (β: −0.037, 95% CI: (−0.063, −0.009), *p* < 0.05), MHH (β: −0.222, 95% CI: (−0.446, −0.156), *p* < 0.05), and MZP (β: −0.030, 95% CI: (−0.079, −0.003), *p* < 0.05) were negatively related to sleep duration. The above results are consistent with the model without covariates adjustment, except for BPH. After adjusting for covariates, BPH demonstrated a significantly negative correlation with sleep duration (β: −0.040, 95% CI: (−0.071, −0.006), *p* < 0.05).

**Figure 3:**
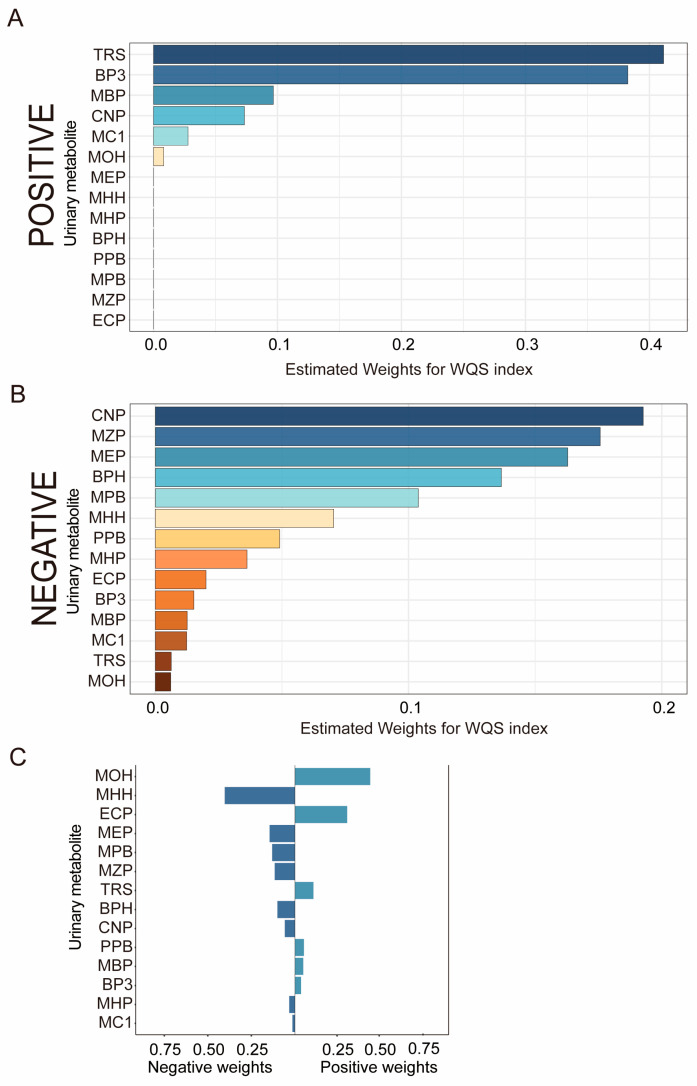
**Estimated weight of urine EDCs mixed exposure with sleep duration in the WQS and QGC models.** Model adjusted for urinary creatinine, age, gender, race, education level, marital status, family-income-to-poverty ratio, physical activity, BMI, smoking status, and alcohol consumption. (**A**) Positive direction of the WQS model. (**B**) Negative direction of the WQS model. (**C**) Each weight represents the proportion of the positive or negative partial impact per individual EDC. The length of each bar indicates the effect size of each exposure in the same direction. Each color in WQS represents a different metabolite of EDCs. The same colors in the QGC model represent the same orientation.

We also employed the WQS and QGC models to examine the relationship between mixed exposure to EDCs and sleep duration (Figure 3). In the WQS model, a positive association was observed, with TRS having the highest weight (41.1%), while in the negative direction, Mono (carboxyisononyl) phthalate (CNP) (19.3%), MZP (17.6%), MEP (16.3%), BPH (13.7%), MPB (10.4%), and MHH (7.0%) were the primary contributors, with CNP (19.3%) significantly negatively impacting sleep duration. The results from the QGC model were generally aligned with those of the WQS model. MOH and TRS were positively associated with sleep duration, whereas MHH, MEP, MZP, and BPH showed negative associations (*p*< 0.05). Notably, MEP, TRS, and MZP all played a role in sleep duration, regardless of whether the exposure was to a single substance or a mixture of multiple substances and whether or not covariates were taken into account. These findings were statistically significant.

### 3.4. Correlation between Urinary EDC Metabolites and Vitamin D Deficiency

The results in Table 3 demonstrate that BP3 (OR: 0.859, 95% CI: (0.813, 0.906)), TRS (OR: 0.954, 95% CI: (0.904, 0.997)), and CNP (OR: 0.833, 95% CI: (0.751, 0.925)) were significantly different from Mono-(3-carboxypropyl) phthalate (MC1) (OR: 0.864, 95% CI: (0.787, 0.949)) (*p* < 0.05), with or without adjustment for covariates. The risk of vitamin D deficiency decreased with increasing exposure to BP3, TRS, CNP, and MC1. Without covariate adjustment, the risk of vitamin D deficiency increased with increasing exposure to MZP. In contrast, with adjusted covariates, MEP was identified as a possible risk factor for vitamin D deficiency.

The results of mixed exposure-adjusted covariates indicated that BP3 (OR: 0.857, 95% CI: (0.810, 0.906)), TRS (OR: 0.931, 95% CI: (0.882, 0.982)), and MC1 (OR: 0.910, 95% CI: (0.825, 0.992)) played a protective role against vitamin D deficiency. Meanwhile, MEP (OR: 1.080, 95% CI: (1.007, 1.158)) and MZP (OR: 1.059, 95% CI: (1.009, 1.133)) (*p* < 0.05) were possible risk factors. The above results were also applicable in the model without adjusting for covariates. Additionally, after adjusting for covariates, MOH and CNP were found to be possible vitamin D deficiency protective factors (*p*< 0.05), while MHH was a risk factor for vitamin D deficiency. All these results were statistically significant.

**Figure 4:**
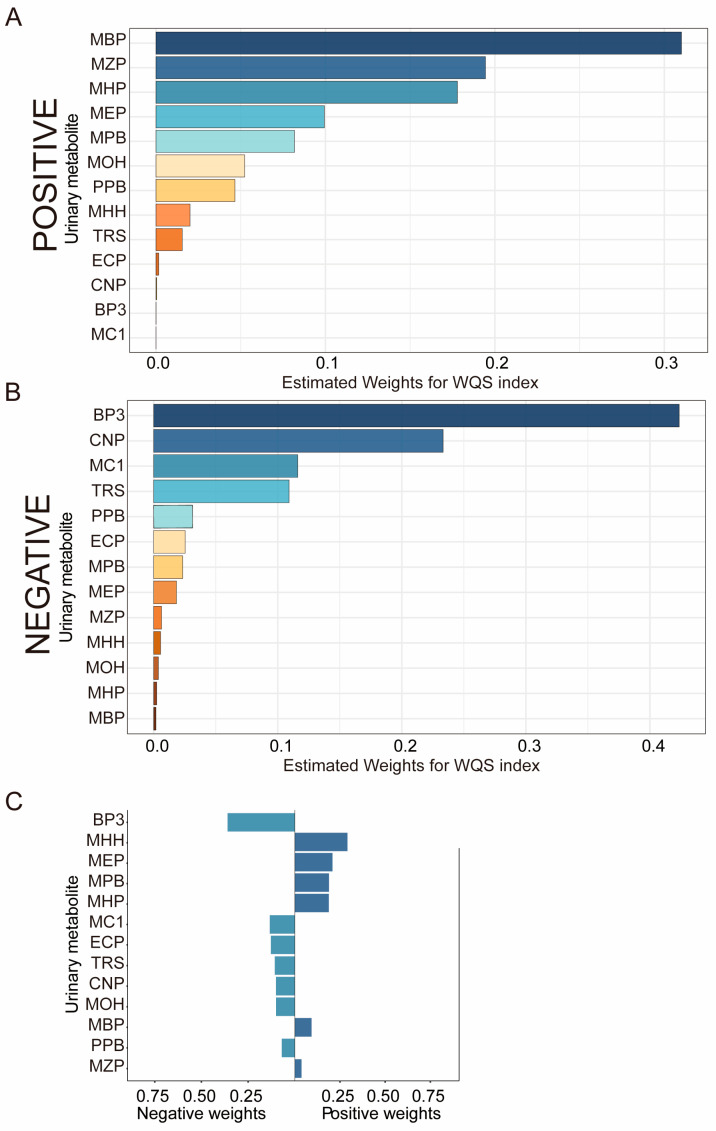
**Estimated weight of urine EDCs mixed exposure with Vitamin D concentrations in the WQS and QGC models.** Model adjusted for urinary creatinine, age, gender, race, education level, marital status, family-income-to-poverty ratio, physical activity, BMI, smoking status, and alcohol consumption. (**A**) Positive direction of the WQS model, (**B**) Negative direction of the WQS model. (**C**) Each weight represents the proportion of the positive or negative partial impact per individual EDCs. The length of each bar indicates the effect size of each exposure in the same direction. Each color in WQS represents a different metabolite of EDCs. The same colors in the QGC model represent the same orientation.

The results of the WQS and QGC models indicated that MHH, MEP, and MZP were risk factors for vitamin D deficiency in mixed exposures to EDCs (Figure 4), while BP3, MC1, and TRS were protective factors for vitamin D deficiency. BP3 contributed the most weight in the mixed exposures. Also, BP3, MC1, and TRS were found effective in single-substance exposures irrespective of covariate adjustment. These results were statistically significant for mixed substances. After adjustment for covariates, only CNP and MEP were affected versus mixed exposures to vitamin D (*p*< 0.05).

### 3.5. Stratified Analysis by Vitamin D Level

We screened substances that were associated with sleep duration and vitamin D in EDCs alone and mixed exposure. The participants were grouped according to vitamin D deficiency or normal level. The results showed that after adjusting for covariates in the vitamin D deficiency group (Table 4), TRS exposure (β: 0.121, 95% CI: (0.023, 0.177), *p* < 0.05) was positively associated with sleep time, whereas MEP exposure (β: −0.052, 95% CI: (−0.077, −0.023), *p* < 0.05) was negatively correlated with sleep time in the vitamin D-normal group. However, this correlation was not statistically significant in the vitamin D-deficient group.

**Figure 5:**
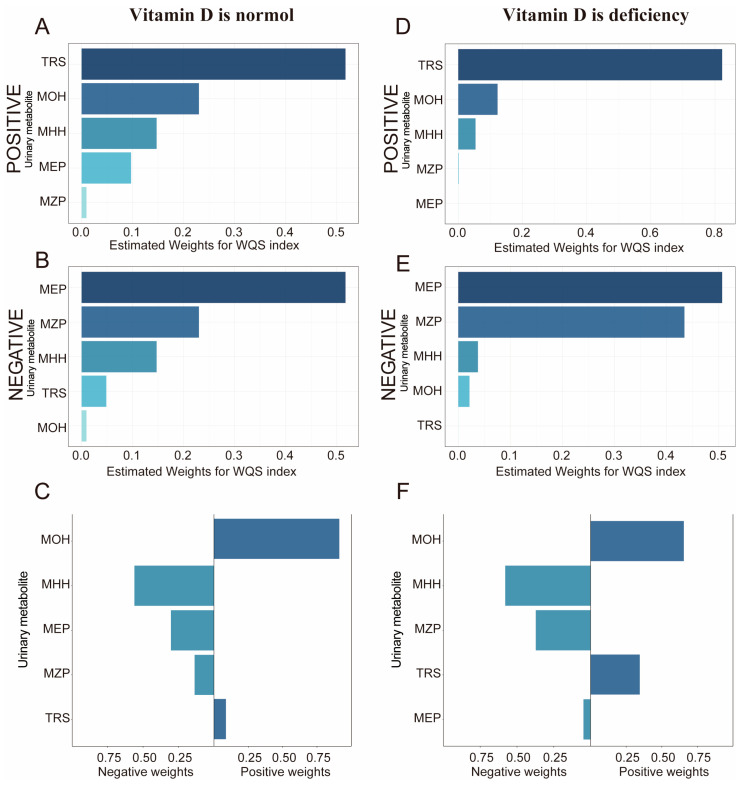
**Estimated weight of EDC mixed exposure with sleep duration in the WQS and QGC models (grouped by vitamin D level).** Model adjusted for age, gender, race, education level, marital status, family-income-to-poverty ratio, physical activity, BMI, smoking status, alcohol consumption, and urinary creatinine. (**A**–**C**) is the WQS and QGC model of the vitamin D non-deficiency group, and (**D**–**F**) is the WQS and QGC model of the vitamin D deficiency group. (**A**) WQS model positive direction, (**B**) WQS model negative direction, and (**C**) QGC model. (**D**) WQS model positive direction, (**E**) WQS model negative direction, and (**F**) QGC model.

The results of TRS and MEP in mixed exposures were generally consistent with those of single EDC exposures (Supplementary Table S4). The results of mixed exposures revealed that MHH and MOH were statistically significantly associated with sleep duration in the vitamin D-normal group but not in the vitamin D-deficient group. The opposite was observed for MZP, with a not statistically significant association in the vitamin D-normal group. However, MZP was negatively correlated with sleep duration in the vitamin D deficiency group (*p*< 0.05). The results of the WQS and QGC models (Figure 5) showed a stable positive correlation of TRS and MOH with sleep duration and a stable negative correlation of MHH, MZP, and MEP with sleep duration (*p*< 0.05).

**Figure 6:**
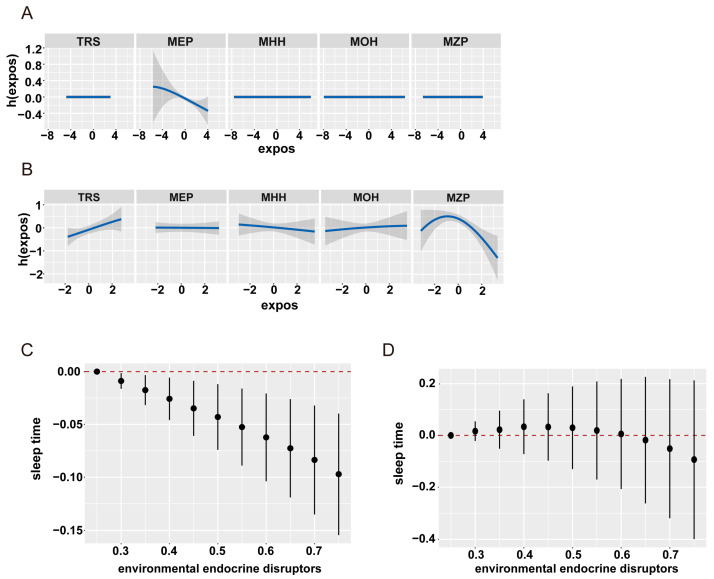
**Association between EDC mixtures and sleep duration in Bayesian kernel machine regression models (grouped by vitamin D level).** Model adjusted for age, gender, race, education level, marital status, family-income-to-poverty ratio, physical activity, BMI, smoking status, alcohol consumption, and urinary creatinine. (**A**,**B**) Univariate exposure–response functions (95%CrI) for single EDCs when other EDCs fixed at 50% percentile values. (**C**,**D**) Overall associations of the mixture of EDCs metabolites on sleep duration in Bayesian kernel machine regression (BKMR). Dots indicate the β value, and vertical lines indicate the 95% credible intervals (CrI).

**Figure 7:**
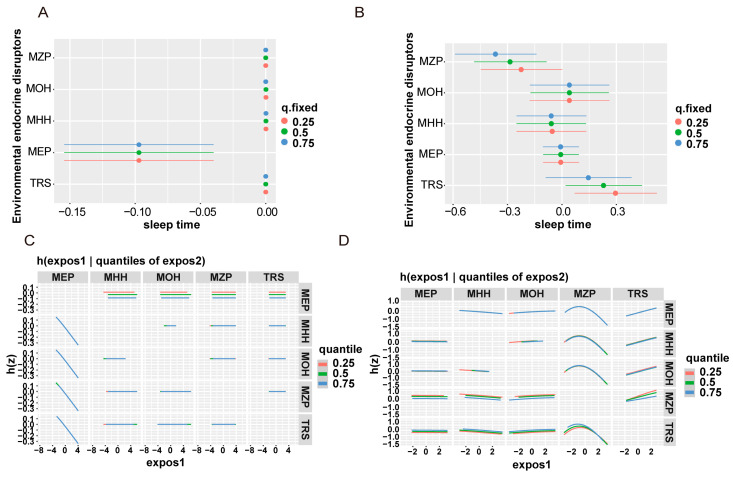
**Association between EDC mixtures and sleep duration in Bayesian kernel machine regression models (grouped by vitamin D level).** (**A**,**B**) describe the estimated difference in sleep time for each EDC from the 25th to the 75th percentile, when all other EDCs are fixed at the 25th (red line), 50th (green line), or 75th percentile (blue line). Dots indicate the estimate, and horizontal lines indicate the 95% credible intervals (CrI). (**C**,**D**) is a bivariate exposure–response function of environmental endocrine disruptors and sleep duration. When an environmental endocrine disruptor is fixed in different (25, 50, 75) percentiles and other EDCs are fixed at the 50th, the average difference between the other EDCs and the sleep duration as a bivariate exposure–response function.

Subsequently, the results of BKMR were examined for univariate exposure–response, bivariate exposure–response, and overall effect plots. The trends of the five EDCs are shown in Figure 6. In the normal vitamin D group, only MEP was associated with sleep duration, whereas in the vitamin D-deficient group, all five EDCs showed an exposure–response trend with sleep duration. We then explored the interactions between EDCs by fixing EDCs at the 25th, 50th, and 75th percentile levels and their dose–response relationship with sleep duration (Figure 7). The results showed that all EDCs had interactions.

## 4. Discussion

This study is the first to use several statistical models and data from the NHANES database to analyze the effects of combined environmental EDC exposures on sleep deficit and to investigate the influence of vitamin D insufficiency levels on this connection. These analyses showed a relationship between individual sleep duration and exposure to MEP, TRS, and MZP alone or in combination. Low vitamin D levels were also linked to BP3, MC1, and TRS. Vitamin D deficiency was associated with longer sleep duration in those exposed to MZP and TRS. Overall, the findings show that exposure to environmental EDCs can affect how long people sleep and that getting enough vitamin D may help lessen the adverse effects of these EDCs on sleep quality.

Environmental chemicals can have complex complementary, overlapping, and additive effects [38], and hundreds of different chemical exposures may begin even at the fetal stages of development [39]. Therefore, it is necessary to determine the exposure risk of mixed endocrine disruptors and any plausible processes influencing individual sensitivity. The statistical models of WQS, QGC, and BKMR have been widely used to assess the effects of chemical combinations on humans. Relative to traditional models, these models can more effectively simulate exposures to chemical mixtures under realistic environmental conditions [40], allowing for the identification of critical compounds within a given mixture. A study conducted on a group of teenagers from Mexico discovered that exposure to higher levels of EDCs may be linked to both later and longer sleep durations, with varying EDCs having a role in both effects [18]. This Mexican study found that Triclosan, Bisphenol A, Mono-benzyl phthalate, Mono-(2-ethyl-5-oxohexyl) phthalate, Mono- (2-ethyl-5-hydroxyhexyl) phthalate, and Mono-ethyl phthalate all significantly contributed to the effect of mixed EDC exposures on sleep. The strong correlations between these substances may explain variances in correlations due to changes in drug levels. The BKMR data further confirmed the strong interactions among various environmental EDCs.

Humans are exposed to a wide variety of EDCs [41] every day through various routes, interfering with normal hormone signaling by affecting endocrine hormone synthesis and competing for hormone-receptor binding, disrupting normal immunity, metabolism, and sleep [42]. Unlike MEP and Mono-n-butyl phthalate (MBP), TRS levels showed a positive correlation with sleep duration in this study. The data partially align with findings from a previous study showing a correlation between elevated levels of certain phthalates and reduced sleep duration in adolescents [43]. Animal studies have demonstrated that exposure to bisphenol A before birth negatively impacts brain volume in children and young rats, as observed using magnetic resonance imaging (MRI) [44]. The suprachiasmatic nucleus (SCN) in the hypothalamus is crucial for regulating circadian rhythms and is highly susceptible to external environmental influences [45]. Research has shown that EDC can disrupt the SCN and cause animal circadian rhythm disturbances. This mechanism involves both endocrine hormones and genes that regulate circadian rhythms [46].

In addition, EDCs can induce metabolic disorders and produce related adverse health effects. The results of this study also found that Benzophenone-3, Mono-(3-carboxy propyl) phthalate, and triclosan were found to be associated with vitamin D deficiency both individually and in combination, in line with the results of a prior Korean study [47]. A previous study from the US also found repeated measures of phthalate metabolites to be negatively correlated with total blood 1,25(OH)_2_D_3_ levels [48]. The interaction between EDCs and vitamin D is mutual. Vitamin D has been shown to reduce the incidence and severity of EDC-induced diseases [49]. Bisphenol A-treated mice exhibit elevated genes associated with vitamin D metabolism, as per studies [50]. An essential function of vitamin D is in metabolism. Following vitamin D administration, mice exposed to BPA showed improvements in their heart, kidney, lung, and neurobehavioral problems [51].

The present results revealed that vitamin D-deficient individuals slept less than people with normal levels. A prior meta-analysis also explored the association between vitamin D levels and sleep disorders [52], ultimately finding vitamin D deficiency related to sleepiness, poorer sleep quality, and shorter sleep duration. Vitamin D deficiency was not associated with sleeping time when using triclosan or monobenzylphthalate. Even so, they were linked to the length of sleep for those who were diagnosed as vitamin D deficient; in contrast, this was not the case with mono-ethyl, mono-(2-ethyl-5-oxohexyl), or mono-(2-ethyl-5-hydroxyhexyl) phthalates. Sleep patterns can be directly and indirectly regulated by vitamin D [53]. Vitamin D receptors can be detected in brain regions involved in sleep regulation [54]. Research has shown variations in the magnetic resonance spectroscopy of the hippocampus in adult mice with vitamin D deprivation, leading to impaired brain amyloid plaque load and astrocyte numbers [55]. Animal tests have shown that mice with vitamin D deficiency exhibit an increased inclination towards sleep, which is associated with circadian rhythm disturbances in mice [56].

Sleep duration is defined as the time during which sleep occurs [57], and it can be impacted by environmental, behavioral, psychological, and pathophysiological factors. Inappropriate sleep duration can contribute to adverse outcomes. Numerous studies have shown the crucial role of sleep in mental health in recent years. The likelihood of developing depression doubles when one is sleep deprived [58]. People with sleep disorders had a 1.65 times higher risk of cognitive impairment compared to those without sleep problems, with a 95% confidence interval of 1.45–1.86 [59]. In the United States, approximately 2–5% of the population has severe depression, while up to 20% experience less severe types of psychiatric disorders [60]. Given the substantial social burden and financial costs associated with psychiatric diseases, prevention is imperative. Our study investigates the relationship between EDCs and sleep and the possible advantages of vitamin D supplementation in this regard. The results offer novel approaches to the prevention and treatment of EDCs that exacerbate existing psychiatric illnesses.

This study has two major strengths. The study population is large and representative, as it was conducted using the NHANES dataset. Various models analyzed the connections between EDC exposure and health effects in American adults, resulting in consistent results. However, these findings are limited by several constraints. The NHANES study is cross-sectional, so it is difficult to establish a causal relationship, highlighting the necessity for future cohort studies that mainly investigate the relationship between EDCs and health outcomes. In the future, we can also explore the molecular mechanism behind it by conducting relevant animal research. Vitamin D is a prevalent vitamin associated with sunlight and exercise patterns. Insufficient data has prevented the analysis of these factors, which may create a bias in the results. In addition, Vitamin D deficiency is defined at different levels in different countries, thus limiting the results from being extended worldwide. Because sleep-disorder questionnaires are inherently subjective, missing data and inconsistencies limit the conclusions that could be drawn. In the future, wearable devices could be used to detect participants’ sleep more objectively and accurately, making the results more powerful. Finally, there may have been bias in these results due to the replacement of EDC levels below the LOD by dividing the LOD by the square root of two [61].

## 5. Conclusions

The current findings provide additional data indicating a potential link between exposure to environmental endocrine disruptors and sleep duration. Vitamin D insufficiency may also worsen the adverse effects of poor sleep caused by exposure to environmental endocrine disruptors.

## Figures and Tables

**Table 1:** Characteristics of the study population.

Characteristics	Total
Gender n(%)	
Male	2709 (48.8)
Female	2767 (51.2)
Age n (%)	
20–39 years	1856 (36.1)
40–59 years	1919 (40.2)
60–80 years	1701 (23.7)
Race n (%)	
Mexican American	767 (7.8)
Other Hispanic	536 (5.5)
Non-Hispanic White	2560 (70.4)
Non-Hispanic Black	1095 (10.2)
Other Race—Including Multi-Racial	518 (6.1)
Educational level n (%)	
Less Than 9th Grade	510 (5.2)
9–11th Grade (Includes 12th grade with no diploma)	808 (11.2)
High School Grad/GED or Equivalent	1276 (22.6)
Some College or AA degree	1574 (30.8)
College Graduate or above	1308 (30.3)
Marital Status n (%)	
Married	2882 (57.1)
Widowed	432 (5.6)
Divorced	597 (10.2)
Separated	164 (2.0)
Never married	995 (18.0)
Living with partner	406 (7.1)
Ratio of family income to poverty n (%)	
<1.3	1751 (21.3)
1.3–3.5	1966 (34.6)
>3.5	1759 (44.1)
Smoke n (%)	
Yes	2473 (44.3)
No	3003 (55.7)
Alcohol drinking n (%)	
Yes	4042 (78.7)
No	1434 (21.3)
Recreational activities n(%)	
Yes	2636 (54.6)
No	2840 (45.4)
Body mass index (BMI) n (%)	
<18.5 kg/m^2^	82 (1.4)
18.5 to <25 kg/m^2^	1509 (28.8)
25 to <30 kg/m^2^	1829 (33.4)
≥30 kg/m^2^	2056 (36.4)
Sleep duration (mean ± SD) (h)	6.87 ± 1.34
Is vitamin D deficient? n (%)	
Yes	5006 (96.0)
No	470 (4.0)

**Table 2:** Correlation between single metabolite and multiple metabolite exposure and sleep time.

Chemicals.	Model 1		Model 2	
	β (95%CI)	*p*	β (95%CI)	*p*
Single substance				
CNP	−0.004 (−0.046, 0.033)	0.760	−0.014 (−0.061, 0.018)	0.288
ECP	0.003 (−0.033, 0.043)	0.808	−0.012 (−0.057, 0.021)	0.366
MBP	−0.012 (−0.056, 0.022)	0.389	−0.024 (−0.075, 0.005)	0.087
MC1	−0.009 (−0.046, 0.024)	0.528	−0.016 (−0.056, 0.013)	0.230
MEP	**−0.039 (−0.063, −0.012)**	**0.004**	**−0.050 (−0.073, −0.022)**	**<0.001**
MHH	−0.016 (−0.057, 0.015)	0.248	−0.026 (−0.071, 0.001)	0.055
MHP	−0.009 (−0.048, 0.024)	0.509	−0.020 (−0.063, 0.008)	0.131
MOH	−0.002 (−0.041, 0.035)	0.891	−0.013 (−0.056, 0.020)	0.350
MZP	**−0.038 (−0.089, −0.016)**	**0.005**	**−0.034 (−0.085, −0.010)**	**0.013**
BP3	−0.003 (−0.021, 0.017)	0.821	**0.028 (0.001, 0.035)**	**0.040**
BPH	**−0.031 (−0.091, −0.008)**	**0.020**	**−0.040 (−0.104, −0.020)**	**0.004**
TRS	**0.046 (0.015, 0.054)**	**0.001**	**0.027 (0.000, 0.040)**	**0.047**
MPB	−0.009 (−0.029, 0.015)	0.523	**−0.041 (−0.058, −0.010)**	**0.005**
PPB	0.001 (−0.016, 0.017)	0.963	**−0.030 (−0.037, 0.000)**	**0.045**
Multi-material				
CNP	−0.013 (−0.066, 0.028)	0.424	−0.014 (−0.068, 0.025)	0.371
ECP	−0.023 (−0.071, 0.006)	0.819	0.031 (−0.052, 0.140)	0.369
MBP	−0.009 (−0.058, 0.031)	0.548	−0.016 (−0.069, 0.021)	0.291
MC1	−0.008 (−0.052, 0.032)	0.637	−0.007 (−0.052, 0.032)	0.645
MEP	**−0.035 (−0.060, −0.007)**	**0.015**	**−0.037 (−0.063, −0.009)**	**0.009**
MHH	**−0.237 (−0.465, −0.175)**	**<0.001**	**−0.222 (−0.446, −0.156)**	**<0.001**
MHP	0.005 (−0.031, 0.044)	0.728	−0.003 (−0.042, 0.033)	0.819
MOH	**0.177 (0.095, 0.412)**	**0.002**	**0.186 (0.108, 0.424)**	**0.001**
MZP	**−0.059 (−0.181, −0.001)**	**0.039**	**−0.030 (−0.079, −0.003)**	**0.033**
BP3	−0.003 (−0.021, 0.017)	0.844	0.022 (−0.003, 0.032)	0.107
BPH	−0.040 (−0.071, 0.006)	0.049	**−0.040 (−0.071, −0.006)**	**0.049**
TRS	**0.043 (0.012, 0.052)**	**0.002**	**0.029 (0.001, 0.041)**	**0.038**
MPB	−0.021 (−0.056, 0.022)	0.382	−0.023 (−0.079, 0.008)	0.113
PPB	0.030 (−0.011, 0.048)	0.216	0.013 (−0.022, 0.038)	0.586

**Note: Model 1**: rough model. **Model 2**: adjusted according to age, gender, race, education level, marital status, family-income-to-poverty ratio, BMI, physical activity, smoking status, alcohol consumption, and urinary creatinine. The thickening part indicates that the *p* < 0.05.

**Table 3:** Correlation between single metabolite and multi-metabolite exposure and vitamin D deficiency.

Chemicals	Model 1		Model 2	
	OR (95%CI)	*p*	OR (95%CI)	*p*
Single substance				
BP3	**0.833 (0.793, 0.874)**	**<0.001**	**0.859 (0.813, 0.906)**	**<0.001**
TRS	**0.909 (0.863, 0.957)**	**<0.001**	**0.954 (0.904, 0.997)**	**0.049**
MPB	1.007 (0.954, 1.064)	0.799	1.023 (0.962, 1.087)	0.464
PPB	0.990 (0.950, 1.032)	0.647	0.991 (0.945, 1.040)	0.716
CNP	**0.805 (0.726, 0.892)**	**<0.001**	**0.833 (0.751, 0.925)**	**0.001**
ECP	1.094 (0.995, 1.203)	0.062	0.959 (0.867, 1.060)	0.412
MBP	1.044 (0.946, 1.151)	0.391	1.043 (0.940, 1.157)	0.428
MC1	**0.844 (0.770, 0.925)**	**<0.001**	**0.864 (0.787, 0.949)**	**0.002**
MEP	1.051 (0.988, 1.119)	0.116	**1.069 (1.002, 1.141)**	**0.044**
MHH	0.964 (0.881, 1.055)	0.425	0.996 (0.908, 1.093)	0.936
MHP	0.991 (0.906, 1.083)	0.840	1.016 (0.928, 1.113)	0.724
MOH	0.938 (0.852, 1.033)	0.196	0.964 (0.873, 1.066)	0.477
MZP	**1.132 (1.032, 1.240)**	**0.008**	1.051 (0.954, 1.158)	0.314
Multi-material				
BP3	**0.834 (0.792, 0.878)**	**<0.001**	**0.857 (0.810, 0.906)**	**<0.001**
TRS	**1.101 (1.000, 1.213)**	**0.042**	**0.931 (0.882, 0.982)**	**0.009**
MPB	0.912 (0.811, 1.025)	0.121	0.957 (0.906, 1.012)	0.122
PPB	0.975 (0.908, 1.047)	0.488	0.940 (0.872, 1.014)	0.112
CNP	0.902 (0.798, 1.020)	0.101	**0.885 (0.780, 0.994)**	**0.047**
ECP	0.890 (0.704, 1.125)	0.330	1.006 (0.772, 1.311)	0.965
MBP	1.019 (0.897, 1.158)	0.769	1.039 (0.912, 1.184)	0.568
MC1	**0.897 (0.800, 0.995)**	**0.032**	**0.910 (0.825, 0.992)**	**0.046**
MEP	**1.139 (1.008, 1.301)**	**0.044**	**1.080 (1.007, 1.158)**	**0.031**
MHH	1.298 (0.891, 1.892)	0.175	**1.453 (1.275, 2.166)**	**0.046**
MHP	1.078 (0.964, 1.207)	0.188	1.121 (0.976, 1.288)	0.107
MOH	0.735 (0.493, 1.096)	0.130	**0.610 (0.399, 0.933)**	**0.023**
MZP	**1.159 (1.042, 1.290)**	**0.007**	**1.059 (1.009, 1.133)**	**0.043**

**Note: Model 1:** rough model. **Model 2:** adjusted according to age, gender, race, education level, marital status, family-income-to-poverty ratio, physical activity, BMI, smoking status, alcohol consumption, and urinary creatinine. The thickening part indicates that the *p* < 0.05.

**Table 4:** The correlation between multi-EDC exposure and sleep duration was grouped by vitamin D level.

Chemicals	Model 1		Model 2	
	β (95%CI)	*p*	β (95%CI)	*p*
Vitamin D is normal				
TRS	**0.038 (0.007,0.048)**	**0.008**	0.023 (−0.004, 0.038)	0.112
MEP	**−0.042 (−0.067, −0.013)**	**0.004**	**−0.050 (−0.075, −0.021)**	**0.001**
MHH	**−0.200 (−0.415, −0.119)**	**<0.001**	**−0.193 (−0.406, −0.109)**	**0.001**
MOH	**0.205 (0.133, 0.447)**	**<0.001**	**0.187 (0.106, 0.422)**	**0.001**
MZP	−0.021 (−0.069, 0.012)	0.173	−0.015 (−0.062, 0.021)	0.332
Vitamin D deficiency				
TRS	**0.135 (0.037, 0.187)**	**0.004**	**0.128 (0.029, 0.183)**	**0.007**
MEP	−0.012 (−0.106, 0.081)	0.797	−0.018 (−0.113, 0.077)	0.709
MHH	−0.246 (−0.809, 0.067)	0.097	−0.266 (−0.842, 0.040)	0.074
MOH	0.242 (−0.080, 0.856)	0.104	0.265 (−0.050, 0.898)	0.079
MZP	−0.145 (−0.345, −0.074)	0.003	−0.143 (−0.349, −0.066)	0.004

**Model 1:** rough model. **Model 2:** adjusted according to age, gender, race, education level, marital status, family-income-to-poverty ratio, physical activity, BMI, smoking status, alcohol consumption, and urinary creatinine. The thickening part indicates that the *p* < 0.05.

## Data Availability

The data were retrieved from publicly available resources and can be accessed from the National Center for Health Statistics of the Center for Disease Control and Prevention at https://www.cdc.gov/nchs/nhanes/index.htm, accessed on 7 April 2024.

## Supplementary Materials

The following supporting information can be downloaded at https://www.mdpi.com/article/10.3390/nu16091291/s1. Figure S1: The correlations of the urinary concentrations in the 15 chemicals examined. Spearman correlation was used to analyze the correlations between the urinary concentrations in the 15 chemicals. The numbers in the lower-left part were the correlation coefficients. The upper-right part was the heat map of the correlation coefficients between chemical concentrations. The white represents the uncorrelation (r = 0.00), the blue represents the positive correlation, and the red represents the negative correlation. The darker the color, the greater the correlation coefficient. Figure S2: The correlations of the urinary concentrations in the 14 chemicals examined. Spearman correlation was used to analyze the correlations between the urinary concentrations in the 14 chemicals. (A) Vitamin D non-deficiency group; (B) Vitamin D deficiency group. The numbers in the lower-left part were the correlation coefficients. The upper-right part was the heat map of the correlation coefficients between chemical concentrations. The white represents the uncorrelation (r = 0.00), the blue represents the positive correlation, and the red represents the negative correlation. The darker the color, the greater the correlation coefficient. Table S1: The distribution of the urinary metabolites in the study population. Table S2: Sleep duration and EDC concentrations grouped by vitamin D level. Table S3: EDCs metabolite distribution in the population (grouped by vitamin D level). Table S4: The correlation between EDC metabolite exposure and sleep duration (grouped by vitamin D level). Table S5: Names of urinary metabolites and their detection limits.
