# Supplementary material for: Vitamin D Deficiency Exacerbates Poor Sleep Outcomes with Endocrine-Disrupting Chemicals Exposure: A Large American Population Study
Source: Nutrients. 2024 Apr 26;16(9):1291. doi: 10.3390/nu16091291 (PMC11085561; doi:10.3390/nu16091291)
Supplement: Supplementary file 1 [file nutrients-16-01291-s001.zip › nutrients-2976859-Supplementary Table S1.pdf]

Table S1. The distribution of the urinary metabolites in the study population

Environmental endocrine disruptors (ng/mL)	Detection rate N (%)	Mean	Percentiles				
			P5	P25	P50	P75	P95
CNP	5272 (96.27)	43.58	0.51	1.15	1.97	3.57	9.59
COP	5404 (98.69)	4.05	1.68	3.76	8.91	28.02	106.57
ECP	5463 (99.76)	27.84	3.79	8.05	14.30	26.86	94.32
MBP	5337 (97.46)	26.62	2.56	7.40	12.79	21.96	53.39
MC1	5215 (95.23)	19.18	0.43	1.03	1.90	3.62	16.64
MEP	5471 (99.91)	4.50	8.24	24.84	62.77	197.19	1096.7
MHH	5445 (99.43)	258.68	2.45	5.08	9.71	18.31	65.99
MHP	3730 (68.12)	18.87	0.37	0.82	1.51	2.90	9.03
MOH	5424 (99.05)	2.90	1.66	3.22	5.74	10.62	35.80
MZP	5358 (97.85)	10.70	1.07	2.97	5.52	11.40	31.40
BP3	5287 (96.55)	10.07	0.66	2.49	7.00	22.45	423.59
BPH	5017 (91.62)	99.83	0.38	0.94	1.63	2.85	8.10
TRS	4143 (75.66)	3.52	0.85	2.26	6.30	26.69	379.67
MPB	5450 (99.53)	61.81	3.25	17.02	73.03	233.05	875.65
PPB	5191 (94.80)	212.88	0.19	1.15	8.16	48.01	199.27
