# Supplementary material for: Vitamin D Deficiency Exacerbates Poor Sleep Outcomes with Endocrine-Disrupting Chemicals Exposure: A Large American Population Study
Source: Nutrients. 2024 Apr 26;16(9):1291. doi: 10.3390/nu16091291 (PMC11085561; doi:10.3390/nu16091291)
Supplement: Supplementary file 1 [file nutrients-16-01291-s001.zip › nutrients-2976859-Supplementary Table S2.pdf]

Table S2. Sleep duration and EDCs concentrations grouped by vitamin D level.

Environmental endocrine disruptors	Non-vitamin d deficiency (mean ± SD)	Vitamin d deficiency (mean ± SD)	χ^2	P-value
Sleep duration	6.89 ± 1.33	6.51 ± 1.57	652048.871	<0.001
BP3	3.22 ± 2.20	2.31 ± 1.87	585115.079	<0.001
BPH	0.51 ± 0.90	0.51 ± 1.00	20755.865	<0.001
TRS	2.63 ± 1.90	2.34 ± 1.85	22153.448	<0.001
MPB	4.03 ± 1.70	4.09 ± 1.71	11530.968	<0.001
PPB	1.98 ± 2.29	1.89 ± 2.22	11600.378	<0.001
CNP	1.00 ± 0.96	0.78 ± 0.93	2366.760	<0.001
ECP	2.79 ± 0.99	2.71 ± 0.92	6335.713	<0.001
MBP	2.39 ± 0.99	2.46 ± 0.89	16565.781	<0.001
MC1	0.99 ± 1.10	0.78 ± 1.10	2898.803	<0.001
MEP	4.02 ± 1.44	4.21 ± 1.46	11067.224	<0.001
MHH	2.35 ± 1.06	2.29 ± 1.00	4.399	0.036
MHP	0.48 ± 1.06	0.46 ± 0.96	37339.190	<0.001
MOH	1.86 ± 1.00	1.78 ± 0.95	166.970	<0.001
BP3	1.59 ± 1.01	1.74 ± 1.03	652048.871	<0.001
