# Supplementary material for: Vitamin D Deficiency Exacerbates Poor Sleep Outcomes with Endocrine-Disrupting Chemicals Exposure: A Large American Population Study
Source: Nutrients. 2024 Apr 26;16(9):1291. doi: 10.3390/nu16091291 (PMC11085561; doi:10.3390/nu16091291)
Supplement: Supplementary file 1 [file nutrients-16-01291-s001.zip › nutrients-2976859-Supplementary Table S3.pdf]

Table S3. EDCs metabolite distribution in the population (grouped by vitamin D level).

Environmental endocrine disruptors(ng/mL)	Detection rate N (%)	Mean	Percentiles				
			P5	P25	P50	P75	P95
**Vitamin D is normal**							
CNP	4822 (96.32)	59.19	0.67	1.37	2.30	4.21	13.51
ECP	4993 (99.74)	32.02	4.48	9.09	15.13	27.99	94.99
MBP	4874 (97.36)	36.46	2.41	7.00	12.00	20.60	50.40
MC1	4764 (95.17)	23.41	0.55	1.26	2.24	4.35	16.60
MEP	5001 (99.90)	6.99	7.96	22.50	55.01	166.18	990.32
MHH	4977 (99.42)	263.15	2.39	5.45	9.60	18.21	67.63
MHP	3372 (67.36)	25.74	0.35	0.79	1.51	2.94	10.38
MOH	4959 (99.06)	3.91	1.65	3.51	5.92	10.82	37.08
MZP	4893 (96.94)	14.80	0.94	2.60	4.91	9.54	26.91
BP3	4831 (96.50)	8.71	0.94	4.08	14.00	69.22	1375.96
BPH	4581 (91.51)	313.78	0.45	0.94	1.57	2.75	7.36
TRS	3781 (75.53)	3.34	1.02	2.89	8.68	44.01	520.06
MPB	4985 (99.58)	90.44	3.92	15.23	68.71	244.89	880.98
PPB	4745 (94.79)	224.47	0.22	1.09	8.15	51.94	267.55
**Vitamin D deficiency**							
CNP	450 (95.74)	4.05	0.51	1.15	1.97	3.57	9.59
ECP	470 (100.00)	26.62	3.79	8.05	14.30	26.86	94.32
MBP	463 (98.51)	19.18	2.56	7.40	12.79	21.96	53.39

MC1	451 (95.96)	4.50	0.43	1.03	1.90	3.62	16.64
MEP	470 (100.00)	258.68	8.24	24.84	62.77	197.19	1096.76
MHH	468 (99.57)	18.87	2.45	5.08	9.71	18.31	65.99
MHP	358 (76.17)	2.90	0.37	0.82	1.51	2.90	9.03
MOH	465 (98.94)	10.70	1.66	3.22	5.74	10.62	35.80
MZP	465 (98.94)	10.07	1.07	2.97	5.52	11.40	31.40
BP3	456 (97.02)	99.83	0.66	2.49	7.00	22.45	423.59
BPH	436 (92.77)	3.52	0.38	0.94	1.63	2.85	8.10
TRS	362 (77.02)	61.81	0.85	2.26	6.30	26.69	379.67
MPB	465 (98.94)	212.88	3.25	17.02	73.03	233.05	875.65
PPB	446 (94.89)	43.58	0.19	1.15	8.16	48.01	199.27
