# Supplementary material for: Vitamin D Deficiency Exacerbates Poor Sleep Outcomes with Endocrine-Disrupting Chemicals Exposure: A Large American Population Study
Source: Nutrients. 2024 Apr 26;16(9):1291. doi: 10.3390/nu16091291 (PMC11085561; doi:10.3390/nu16091291)
Supplement: Supplementary file 1 [file nutrients-16-01291-s001.zip › nutrients-2976859-Supplementary Table S4.pdf]

Table S4. The correlation between EDCs metabolite exposure and sleep duration (grouped by vitamin D level).

Chemicals	Model I		Model II	
	β (95%CI)	P	β (95%CI)	P
**Vitamin D**				
BP3	0.020 (-0.005, 0.031)	0.160	-0.009 (-0.025, 0.014)	0.558
TRS	**0.035 (0.005, 0.046)***	**0.014**	0.017 (-0.008, 0.033)	0.236
MEP	**-0.041(-0.065, -0.013)***	**0.004**	**-0.052 (-0.077, -0.023)***	**<0.001**
**Vitamin D**				
BP3	0.040 (-0.041, 0.106)	0.382	0.024 (-0.059, 0.097)	0.633
TRS	**0.134 (0.036, 0.185)***	**0.004**	**0.121 (0.023, 0.177)***	**0.011**
MEP	-0.009 (-0.102, 0.083)	0.842	-0.014 (-0.110, 0.080)	0.760

Model 1 : Rough model.

Model 2 : Adjusted according to age, gender, race, education level, marital status, family PIR, physical activity, smoking, drinking, BMI and urinary creatinine.

* Means P < 0.05
