# Supplementary material for: Vitamin D Deficiency Exacerbates Poor Sleep Outcomes with Endocrine-Disrupting Chemicals Exposure: A Large American Population Study
Source: Nutrients. 2024 Apr 26;16(9):1291. doi: 10.3390/nu16091291 (PMC11085561; doi:10.3390/nu16091291)
Supplement: Supplementary file 1 [file nutrients-16-01291-s001.zip › nutrients-2976859-Supplementary Table S5.pdf]

Table S5. Names of urinary metabolites and their detection limits.

Abbreviation of variable	Variable name	LOD
URXCNP	Mono(carboxyisononyl) phthalate	0.2
URXCOP	Mono(carboxyisoctyl) phthalate	0.3
URXECP	Mono-2-ethyl-5-carboxypentyl phthalate	0.4
URXMBP	Mono-n-butyl phthalate	0.4
URXMC1	Mono-(3-carboxypropyl) phthalate	0.4
URXMEP	Mono-ethyl phthalate	1.2
URXMHH	Mono-(2-ethyl-5-hydroxyhexyl) phthalate	0.4
URXMHP	Mono-(2-ethyl)-hexyl phthalate	0.8
URXMOH	Mono-(2-ethyl-5-oxohexyl) phthalate	0.2
URXMZP	Mono-benzyl phthalate	0.3
URXBP3	Benzophenone-3	0.4
URXBPH	Bisphenol A	0.2
URXTRS	Triclosan	1.7
URXMPB	Methyl paraben	1.0
URXPPB	Propyl paraben	0.1
