# Supplementary figures and images for: Vitamin D Deficiency Exacerbates Poor Sleep Outcomes with Endocrine-Disrupting Chemicals Exposure: A Large American Population Study
Source: Nutrients. 2024 Apr 26;16(9):1291. doi: 10.3390/nu16091291 (PMC11085561; doi:10.3390/nu16091291)

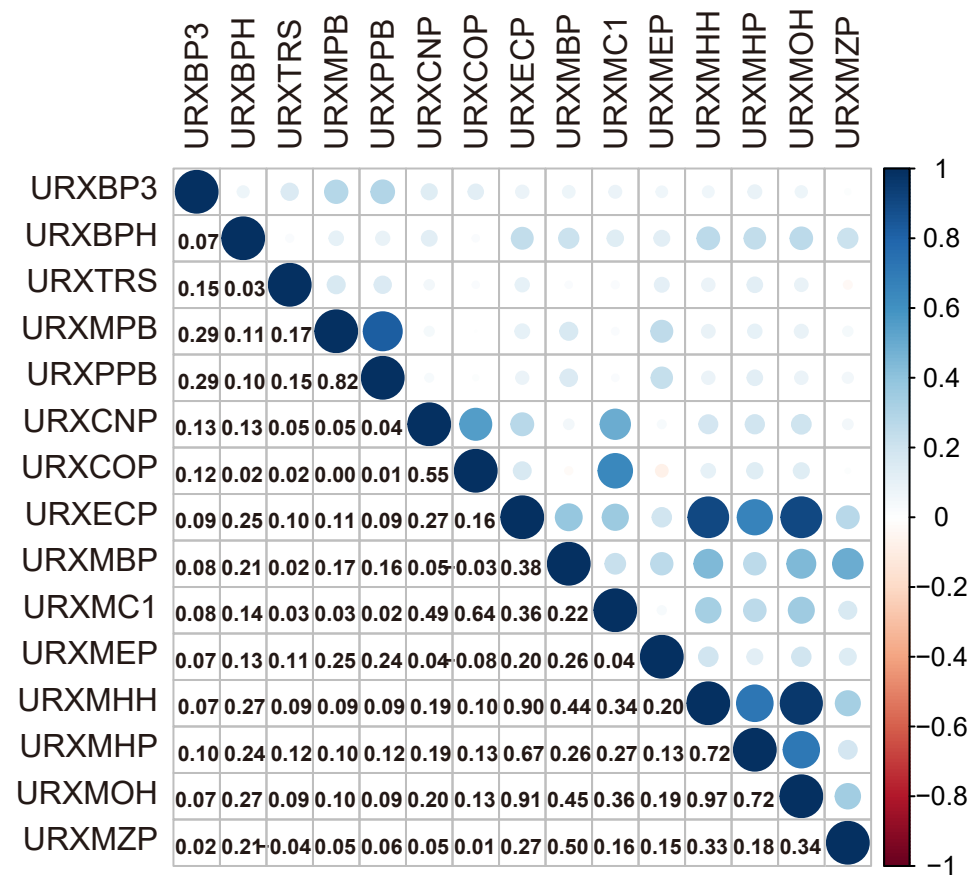

Supplement: Supplementary file 1 [file nutrients-16-01291-s001.zip › nutrients-2976859-Supplementary Figure S1.pdf]

A

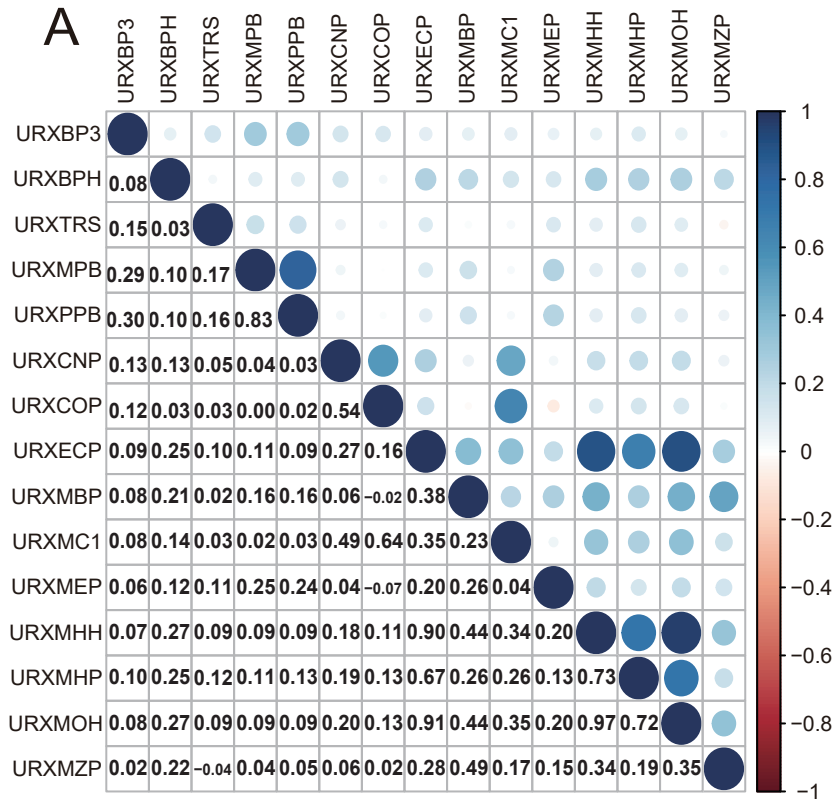

B

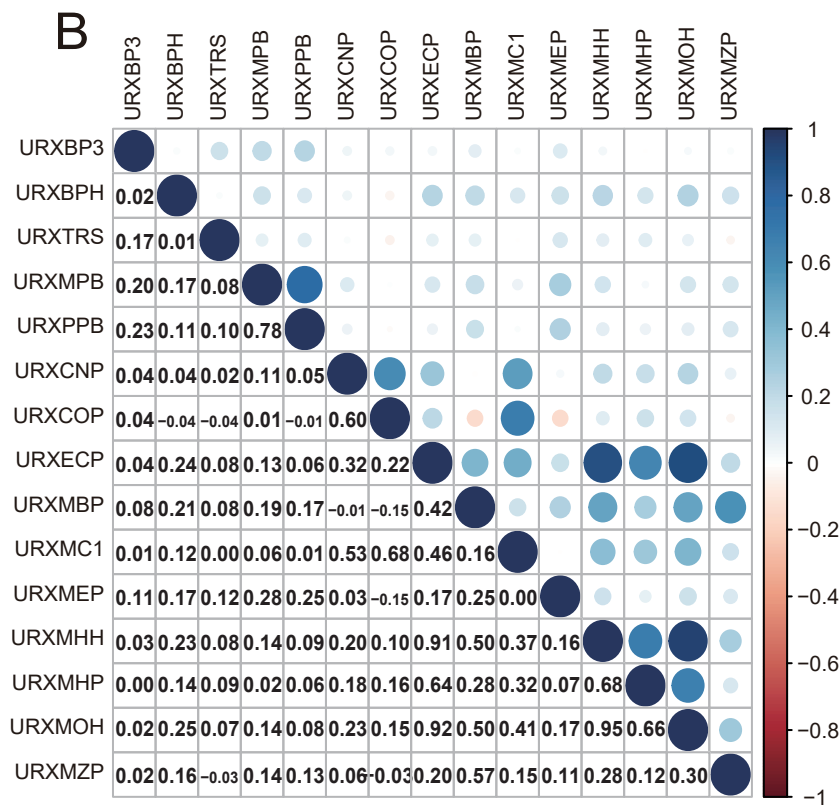

Supplement: Supplementary file 1 [file nutrients-16-01291-s001.zip › nutrients-2976859-Supplementary Figure S2.pdf]
